# Gas Phase Chemical Evolution of Uranium, Aluminum, and Iron Oxides

**DOI:** 10.1038/s41598-018-28674-6

**Published:** 2018-07-11

**Authors:** Batikan Koroglu, Scott Wagnon, Zurong Dai, Jonathan C. Crowhurst, Michael R. Armstrong, David Weisz, Marco Mehl, Joseph M. Zaug, Harry B. Radousky, Timothy P. Rose

**Affiliations:** 1 Physical and Life Sciences Directorate, Lawrence Livermore National Laboratory, Livermore, California 94550 USA; 2 Present Address: Department of Chemistry, Materials, and Chemical Engineering, Politecnico di Milano, Milan, Italy

## Abstract

We use a recently developed plasma-flow reactor to experimentally investigate the formation of oxide nanoparticles from gas phase metal atoms during oxidation, homogeneous nucleation, condensation, and agglomeration processes. Gas phase uranium, aluminum, and iron atoms were cooled from 5000 K to 1000 K over short-time scales (∆t < 30 ms) at atmospheric pressures in the presence of excess oxygen. *In-situ* emission spectroscopy is used to measure the variation in monoxide/atomic emission intensity ratios as a function of temperature and oxygen fugacity. Condensed oxide nanoparticles are collected inside the reactor for *ex-situ* analyses using scanning and transmission electron microscopy (SEM, TEM) to determine their structural compositions and sizes. A chemical kinetics model is also developed to describe the gas phase reactions of iron and aluminum metals. The resulting sizes and forms of the crystalline nanoparticles (FeO-wustite, eta-Al_2_O_3_, UO_2_, and alpha-UO_3_) depend on the thermodynamic properties, kinetically-limited gas phase chemical reactions, and local redox conditions. This work shows the nucleation and growth of metal oxide particles in rapidly-cooling gas is closely coupled to the kinetically-controlled chemical pathways for vapor-phase oxide formation.

## Introduction

Gas phase nucleation and growth of metal oxide nanoparticles is an important topic for many areas of chemistry, physics, material science, and engineering^1–6^. In the field of nuclear forensics, the formation of particles following a nuclear explosion is a special case involving the rapid condensation of material from an initial high-temperature plasma state. If an explosion occurs close to Earth’s surface, surrounding materials will be entrained into the fireball and heterogeneous nucleation processes are expected to dominate, particularly during the late-stages of fallout formation. For explosions that are further from the surface, homogeneous nucleation of particles will dominate, especially during early stage condensation and the vapor pressures of metal oxides derived from major device constituents will play a critical role in determining the condensation process. Understanding the fate and transport of post-detonation radioactive material in the atmosphere requires fundamental data concerning the physics and chemistry of particle formation.

Nuclear debris formation models typically assume mass transport is driven by diffusional mechanisms with equilibrium vapor pressure gradients between the vapor and condensed phases^7–11^. However, inconsistencies are sometimes observed between fractionation measured in debris and predictions based on thermodynamic data. The volatile behavior of uranium is a notable example^12^. Also, the rapid time scales for nuclear fireball cooling can influence the chemical evolution in the gas phase, which affects the nucleation rate and all subsequent physical and chemical processes such as condensation, collisions, coalescence, and transport of particulates. Consequently, understanding the gas phase chemistry of recombination reactions is required to accurately describe the condensation patterns observed during the fast cooling of a nuclear explosion, particularly in situations where homogeneous nucleation processes dominate.

We recently developed a plasma flow reactor that allows *in situ* optical spectroscopy measurements of cooling gas phase molecular species before they form condensed metal oxides^13^. This enables us to monitor the gas phase chemical evolution leading to particle formation. In this study, we compare iron, aluminum, and uranium oxide formation during the homogeneous nucleation and condensation of particles. These three metals are chosen because their oxides exhibit very distinct volatilities^14^. Emission spectroscopy is used to track the high-temperature formation of vapor phase monoxides relative to atomic species. Crystalline nanoparticles were collected and analyzed *ex-situ* using scanning and transmission electron microscopy (SEM, TEM) to determine their sizes and structural phases. In addition, a chemical kinetic model was developed for iron and aluminum to describe the evolution from gas-phase oxidation to particle formation. Our objective is to better understand the interconnection between chemical reactions and micro-physical processes (e.g. nucleation, condensation, growth, etc.) on time scales that are relevant to fallout formation.

## Experimental Setup and Procedure

**Figure 1:**
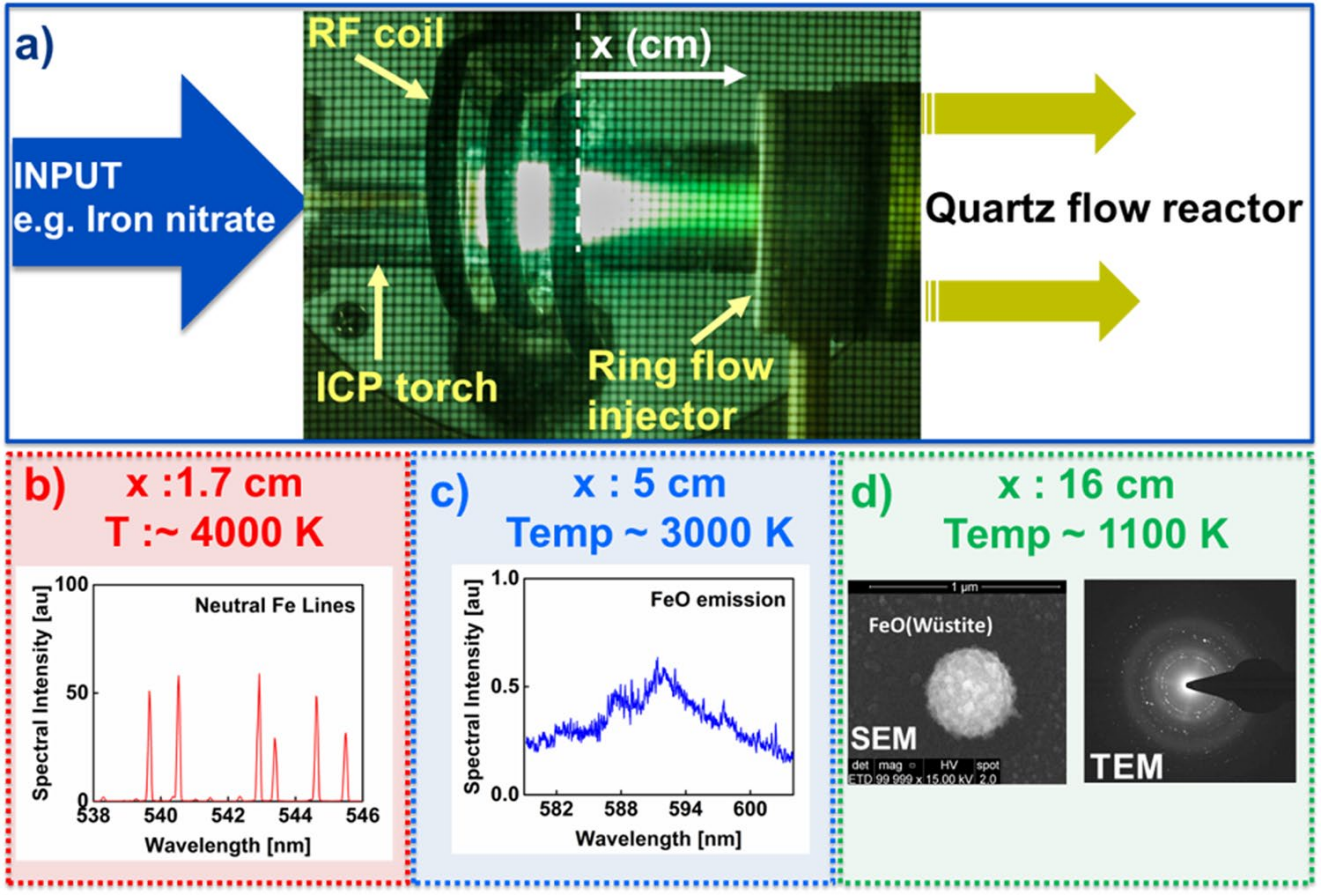
Experimental approach. (**a**) Aqueous solutions of metal salts (e.g. iron nitrate) are input to the plasma flow reactor. *In-situ* line-of-sight optical emission spectra are recorded at locations measured from the end of the RF coil for (**b**) atomic and (**c**) di-atomic species (in this example Fe, and FeO, respectively). (**d**) Oxide particles are collected inside the reactor and analyzed *ex-situ* using electron microscopy. The crystal structures were determined from electron diffraction measurements in a TEM (e.g. FeO (wüstite)).

The plasma flow reactor used in our experiments is described in a recent publication^13^. The details of the experimental approach are illustrated in Fig. 1. The reactor consists of a 20 mm OD inductively coupled plasma (ICP) torch attached to a 40 mm OD quartz tube by means of an adapter (ring flow injector as shown in Fig. 1). Aqueous solutions of Al, Fe, or U nitrate are introduced to the plasma using a nebulizer with argon carrier gas. For this study, the outermost, central, and innermost flow rates through the ICP torch were set to 14.5, 0, and 1 l/min, respectively. The ring flow injector can be used to introduce gas downstream from the plasma, but it was not used in that capacity in these experiments.

The emission spectra of the hot gas were recorded at different locations along the reactor using a grating spectrometer (JY Horiba, HR 460; FWHM spectral resolution = 0.05 nm). A moving stage is vertically positioned next to the plasma flow reactor to accommodate optical components and particle collection probes. A stepper-motor and corresponding Labview code control the stage movement with high precision (+/−0.01 cm), which enable repeatable measurements at the same locations, when studying different analytes.

When the analyte solution (e.g. iron nitrate) passes through the argon plasma, it is decomposed into its constituent elements. After exiting the plasma, the gas cools very quickly and the elements initially recombine to form simple molecules and later condense to form oxide nanoparticles. The temperature distribution along the reactor was measured using optical emission of atomic (Fe) and diatomic (AlO) species. The temperature gradient is steepest in the region immediately downstream from the plasma, decreasing from ~4000 K to 3000 K over a lateral distance of about 3 cm (Fig. 1b,c) and thereafter cools more gradually. Particulates were collected within the reactor at 16 cm from the RF coil, where the gas temperature was about 1100 K (determined by means of a K-type thermocouple). Silicon wafers, carbon, or silicon nitride films were attached to a narrow diameter probe (6 mm OD) inserted from the exhaust side of the quartz tube in order to collect particles. The substrates positioned perpendicular to the flow were taken out of the setup at the end of the experiments and analyzed *ex-situ* using scanning and transmission electron microscopy. Spherical aggregates up to 500 nm in diameter were observed in SEM images (Fig. 1d). Electron diffraction and high resolution TEM images were used to determine the crystal structure and the crystallinity of the oxide particles (e.g. FeO-wüstite in Fig. 1d). The same experimental approach was followed for measurements using uranyl (UO_2_(NO_3_)_2_. 6H_2_O) and aluminum (Al(NO_3_)_3_. 9H_2_O) nitrates.

For the current paper, Fe, Al, and U were investigated separately under identical run conditions. This allowed us to compare differences in the chemical evolution and oxide particle characteristics for each element. The number densities of species input to the plasma-flow reactor are summarized in Table 1. The metal concentrations were kept identical for all six experiments. These concentrations were calculated at room temperature (298 K) and atmospheric pressure based on the peristaltic pump flow rate (0.21 ml/min of aqueous solution), concentrations of aqueous solutions of metal salts, efficiency of the nebulizer (12%), and the argon gas flow rate settings of the digital flow controllers. The concentrations of the iron, uranyl, and aluminum nitrates were 0.1, 0.125, and 0.087 gram of salt per milliliter of water, respectively. In addition, the amount of oxygen is different for the first three and last three experiments given in Table 1. In the first group of experiments (low oxygen fugacity), the only oxygen present was that which was supplied by the aqueous solution. In the second group of experiments (high oxygen fugacity), a small amount of oxygen (50 ml/min) was added to the argon carrier gas using a digital flow controller (Omega engineering, FMA2604A) through the nebulizer line. Note that the oxygen concentration was more than stochiometric even for the low fugacity experiments (experiments 1, 2, and 3). Metal concentrations were kept substantially low in this study in an effort to achieve atom number densities that were reasonably consistent with those of nuclear fireballs.

**Table 1:** The number densities of species input to the plasma-flow reactor.

Experiment number	${\mathop{{\boldsymbol{N}}}\limits^{\bullet }}_{{\boldsymbol{Fe}}}$	${\mathop{{N}}\limits^{\bullet }}_{{U}}$	${\mathop{{\boldsymbol{N}}}\limits^{\bullet }}_{{\boldsymbol{Al}}}$	${\mathop{{\boldsymbol{N}}}\limits^{\bullet }}_{{\boldsymbol{O}}}$	${\mathop{{\boldsymbol{N}}}\limits^{\bullet }}_{{\boldsymbol{H}}}$	${\mathop{{\boldsymbol{N}}}\limits^{\bullet }}_{{\boldsymbol{N}}}$	${\mathop{{\boldsymbol{N}}}\limits^{\bullet }}_{{\boldsymbol{Ar}}}$
I	3.72 × 10^18^	0.00	0.00	9.08 × 10^20^	1.75 × 10^21^	1.12 × 10^19^	3.81 × 10^23^
II	0.00	3.74 × 10^18^	0.00				
III	0.00	0.00	3.49 × 10^18^		1.74 × 10^21^	1.05 × 10^19^	
IV	3.72 × 10^18^	0.00	0.00	2.14 × 10^21^	1.75 × 10^21^	1.12 × 10^19^	
V	0.00	3.74 × 10^18^	0.00				
VI	0.00	0.00	3.49 × 10^18^		1.74 × 10^21^	1.05 × 10^19^	

Units: ${\mathop{N}\limits^{\bullet }}_{species}$[atom/min].

## Results and Discussion

### Temperature Distribution along the Reactor

The electronic and rovibronic spectra of Fe and Al and their monoxides have been extensively studied in the literature^15–19^ and can be used to constrain the temperatures of the vapor phase species at high temperatures. The excitation temperatures obtained from the atomic transitions of iron represent the electron temperatures^20^, whereas rotational temperatures can be determined from the rovibronic transitions of the AlO molecule^21^. We performed temperature measurements using both species (i.e. Fe and AlO). The results (shown in Fig. S1 in the supplementary material) for the two sets of measurements were consistent within the measurement uncertainties, +/−10%. Therefore, these results suggest that local thermodynamic equilibrium was established between different degrees of freedom for different species. This situation is different than the one observed in close proximity of the inductively coupled plasma source^22^ and during laser ablation of iron and aluminum as reported in the literature^23^. A discrepancy between the electronic and rotational temperatures were noted in those experiments.

**Figure 2:**
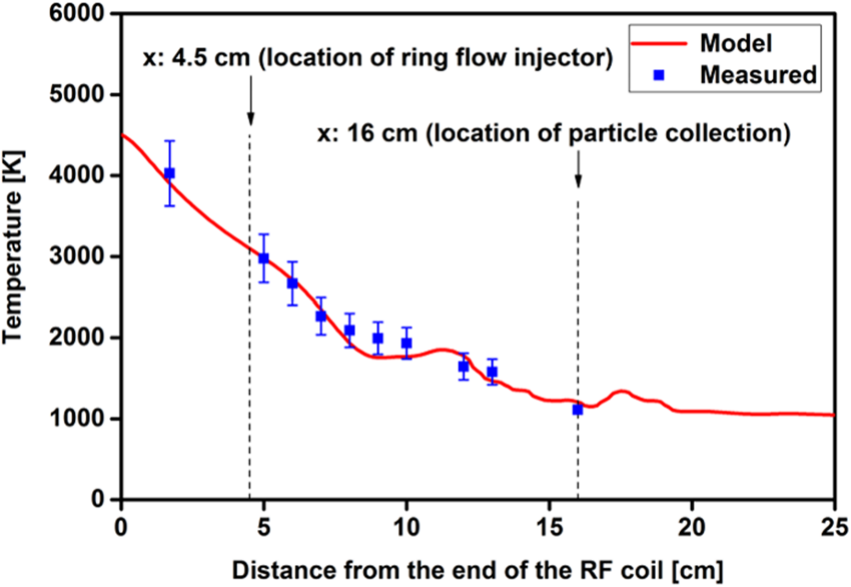
Variation of measured and modeled temperatures as a function of distance along the flow reactor.

A computational fluid dynamics (CFD) simulation of our experimental setup^13^ was used to model the temperature distribution along the reactor. Measured temperatures and CFD model predictions are plotted in Fig. 2. The gas temperature at 16 cm was measured using a thermocouple in the center of the flow field. The temperature predictions along the center line of the reactor axis were extracted from the model and plotted in Fig. 2. The model predictions agreed reasonably well with the measured temperatures. The flow field exhibited eddies after the ring flow injector location (x = 4.5 cm) due to the abrupt increase in reactor diameter from 20 to 40 mm. As a result, small fluctuations in temperature were seen in the model predictions between 5 and 20 cm locations. Note that the plasma flow reactor is a steady state system; therefore, residence time of the species is dependent on the axial distance through the flow velocity. As a result, the CFD model was also used to infer the variation of temperature as a function of time. Temperature vs time and location obtained from the simulations for a parcel of gas injected from the center of the ICP torch is plotted in Fig. S2 in the supplementary material. The cooling time scales were on the order of tens of milliseconds (i.e. ∆t = 27 ms for ∆x = 16 cm). This temperature profile was used to constrain the chemical kinetics simulations described in further detail in section 3.6.

### Iron Oxide results

**Figure 3:**
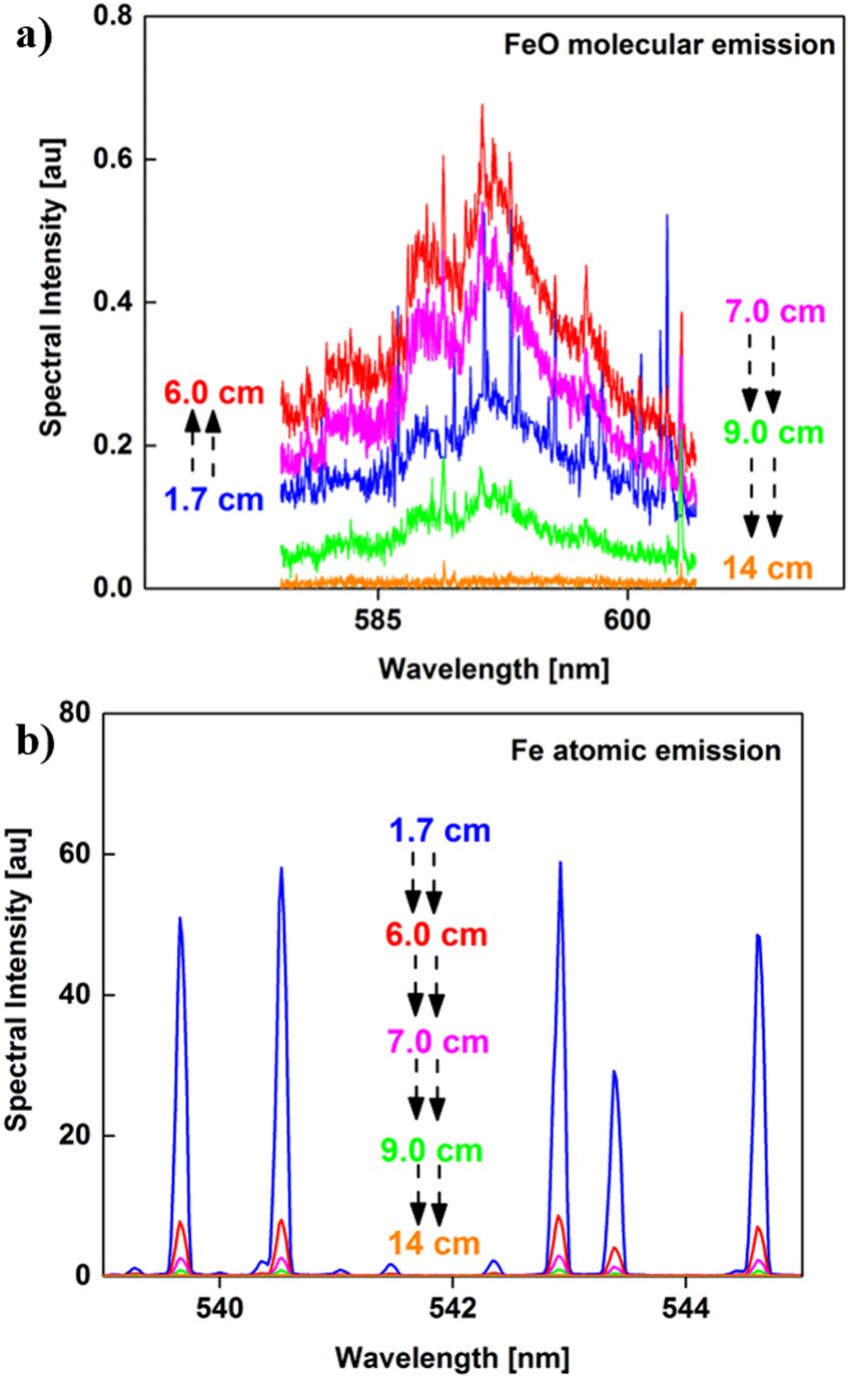
Emission spectra of (**a**) molecular FeO and (**b**) atomic Fe taken at different locations along the reactor. Black arrows are used to show the direction of change in intensity. FeO emission intensity first increases and then decreases, whereas Fe intensity always decreases.

Emission spectra of iron monoxide molecules and neutral iron atoms are shown in Fig. 3(a) and (b), respectively. The spectra were recorded at various positions along the flow reactor axis relative to the position of the RF coil as noted in Fig. 1(a). The broadband emission spectra of FeO increased in intensity between 1.7 and 6 cm, whereas the atomic line intensities decreased. The increase in FeO emission intensity suggests FeO molecules are forming due to the oxidation of Fe atoms with decreasing temperature. Both emission intensities decreased at distances >6 cm.

**Figure 4:**
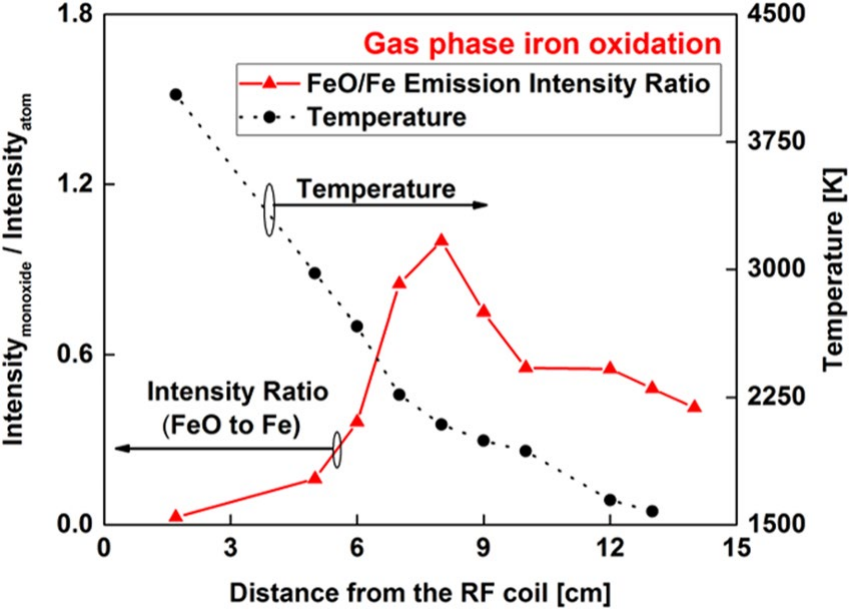
Variation of molecular-to-atomic emission intensity ratio (I_FeO_/I_Fe_) as a function of distance along the flow reactor (left y-axis), showing the chemical evolution from Fe atoms to FeO molecules. Temperature is shown in the right y-axis.

The area underneath the broadband molecular FeO emission was integrated between 579 and 604 nm and divided by the line intensity of the atomic emission at 540.53 nm. This data processing was also pursued by other groups^23,24^ to infer the chemical evolution from atoms to molecules in the gas phase. The variation of molecular-to-atomic emission intensity ratio as a function of distance along the flow reactor is plotted in Fig. 4 for iron. The temperatures calculated from the intensities of iron atoms are shown in the right y-axis of the figure. A sharp inflection in intensity ratio is observed at the 8 cm position, where the temperature was 2100 K. After that point, the relative FeO/Fe intensity decreased, which could be attributed to the formation of a higher iron oxide (e.g. Fe_3_O_4_ or Fe_2_O_3_) or the condensation of iron monoxide vapor. This question can be resolved by determining the composition of the particles that formed.

**Figure 5:**
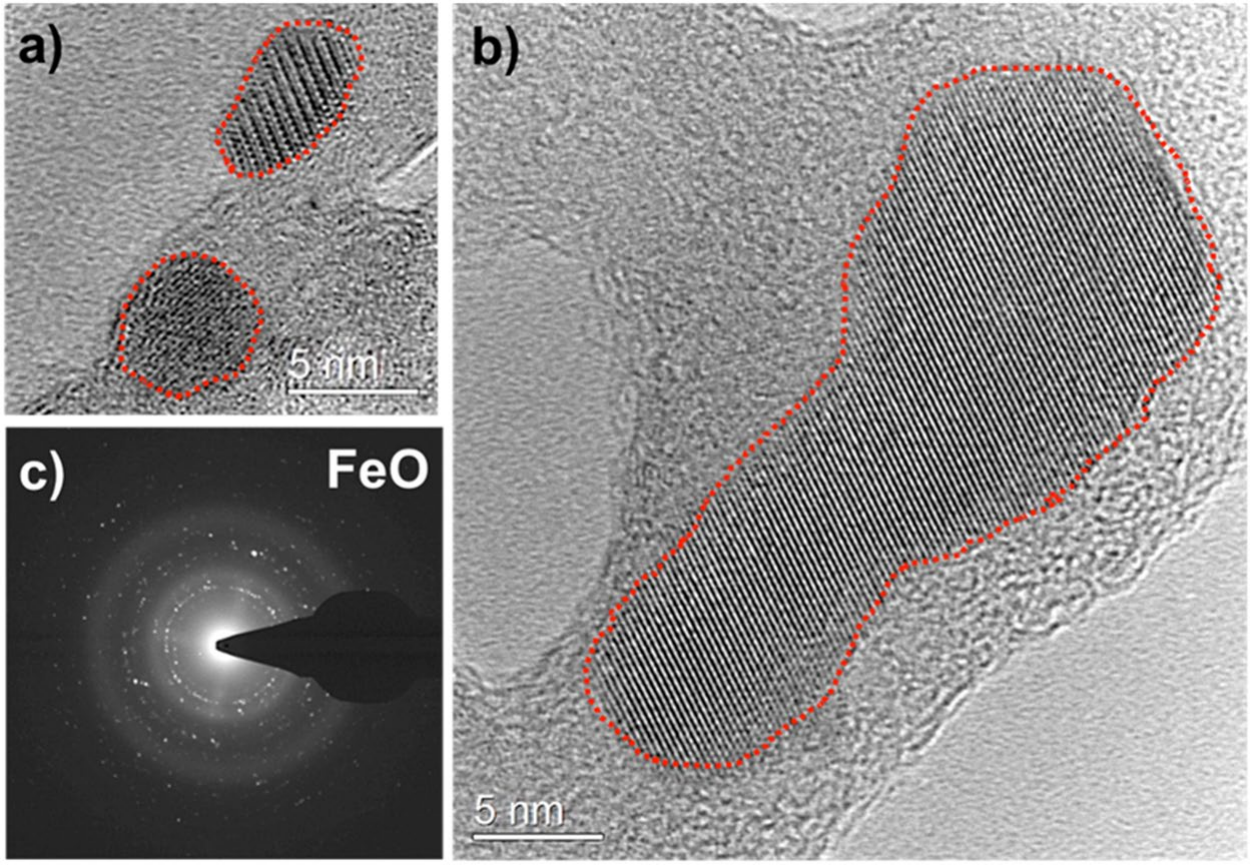
High-resolution TEM images of FeO particles collected on a carbon film. Individual FeO crystals ranging in size from 5 nm to 30 nm are shown in (**a**,**b**), respectively. Red dashed lines are drawn around the ordered structure of crystals to clearly identify the nano-particles. Selected-area electron diffraction pattern is given in (**c**), matching FeO (wustite) crystal structure.

High-resolution TEM images of the iron oxide particles collected on a carbon film are shown in Fig. 5(a,b). The carbon film is positioned at 16 cm from the RF coil, where the gas temperature is about 1100 K. The ordered structure of crystals can be easily discerned and individual particles in the sizes of 5 to 30 nm are observed. A selected-area electron diffraction pattern (SADP) is shown in Fig. 5(c). Iron oxide particles are crystalline and predominant reflections of the SADP match FeO (wüstite). This indicates that the stoichiometry was conserved during the phase change from FeO molecules to its condensates. Chemical equilibrium calculations based on the minimization of the Gibbs free energies were performed using thermochemical properties (enthalpies and entropies) reported in the literature^25^. The results indicated that Fe_2_O_3_ is the thermodynamically favored species for the given experimental conditions (T~1100 K). This suggests the reactions were kinetically limited at these fast cooling rates; the equilibrium-state product, Fe_2_O_3_, did not have sufficient time to form.

We performed the same experiments using a silicon nitride film, which is more chemically stable compared to carbon at these temperatures. Crystalline FeO (wüstite) particles were observed again. The TEM images of iron oxide particles collected on a silicon nitride film are given in the supplementary material (See Fig. S3).

### Aluminum Oxide Results

**Figure 6:**
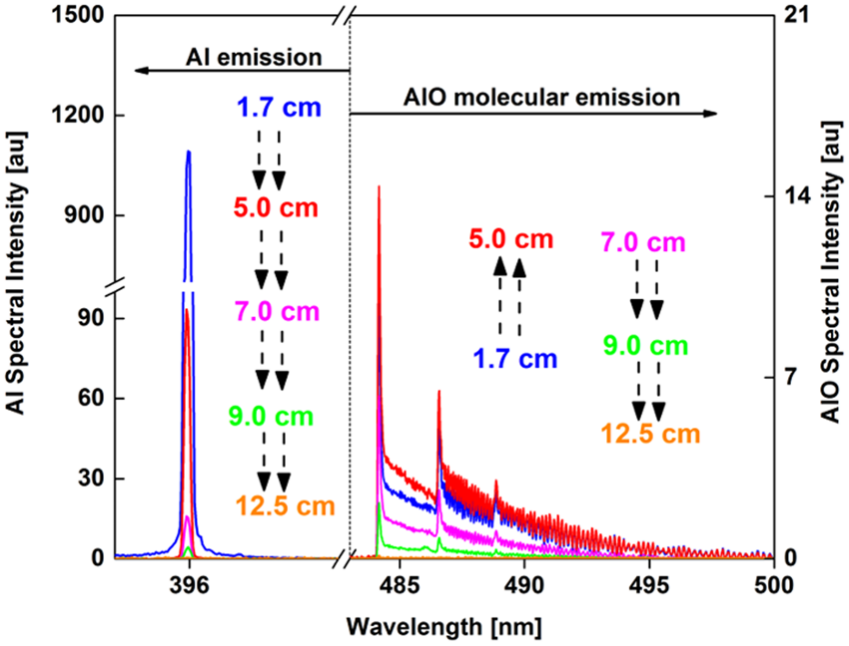
Emission spectra of aluminum (left y-axis) and aluminum monoxide (right y-axis) recorded at different locations along the reactor. Black arrows are used to show the direction of the change in intensity. AlO emission intensity first increases and then decreases, whereas Al emission intensity always decreases as a function of distance along the reactor.

The emission spectra of neutral aluminum atom and aluminum monoxide molecule recorded at various locations along the reactor are shown in Fig. 6. There are multiple AlO rovibronic bands between 440 and 540 nm. High resolution emission spectra of AlO was recorded for the Δν = 0 band of the transition B_2_Σ+ → X_2_Σ+^15^. The broadband emission spectra of AlO increased in intensity between 1.7 and 5 cm locations, whereas the atomic line intensities decreased, indicating the formation of AlO molecules from Al atoms. Both emission intensities decreased further downstream of the reactor (>5 cm).

**Figure 7:**
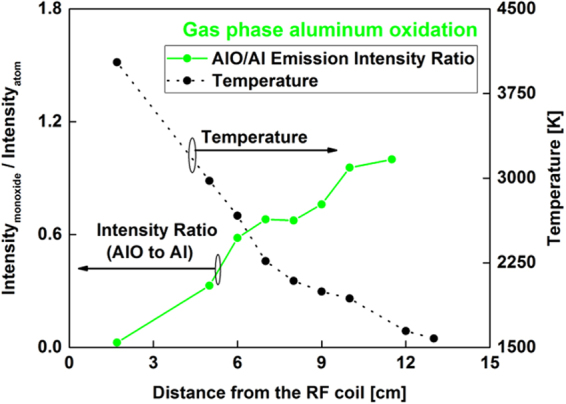
Variation of molecular-to-atomic emission intensity ratio (I_AlO_/I_Al_) as a function of distance along the flow reactor (left y-axis), showing the progressive in-growth of AlO molecules. Temperature is shown in the right y-axis.

The area underneath the AlO emission spectra was integrated between 484 and 507 nm and normalized to the atomic intensity at 396.1 nm. The variation of molecular-to-atomic emission intensity ratio as a function of distance along the flow reactor is given in Fig. 7. The AlO/Al intensity ratio gradually increased with distance along the reactor and did not show an inflection point like that of the FeO/Fe spectra. However, spectra taken at higher oxygen fugacity showed an inflection point as described in section 3.5. These trends are discussed in section 3.6 with the aid of chemical kinetic model predictions.

**Figure 8:**
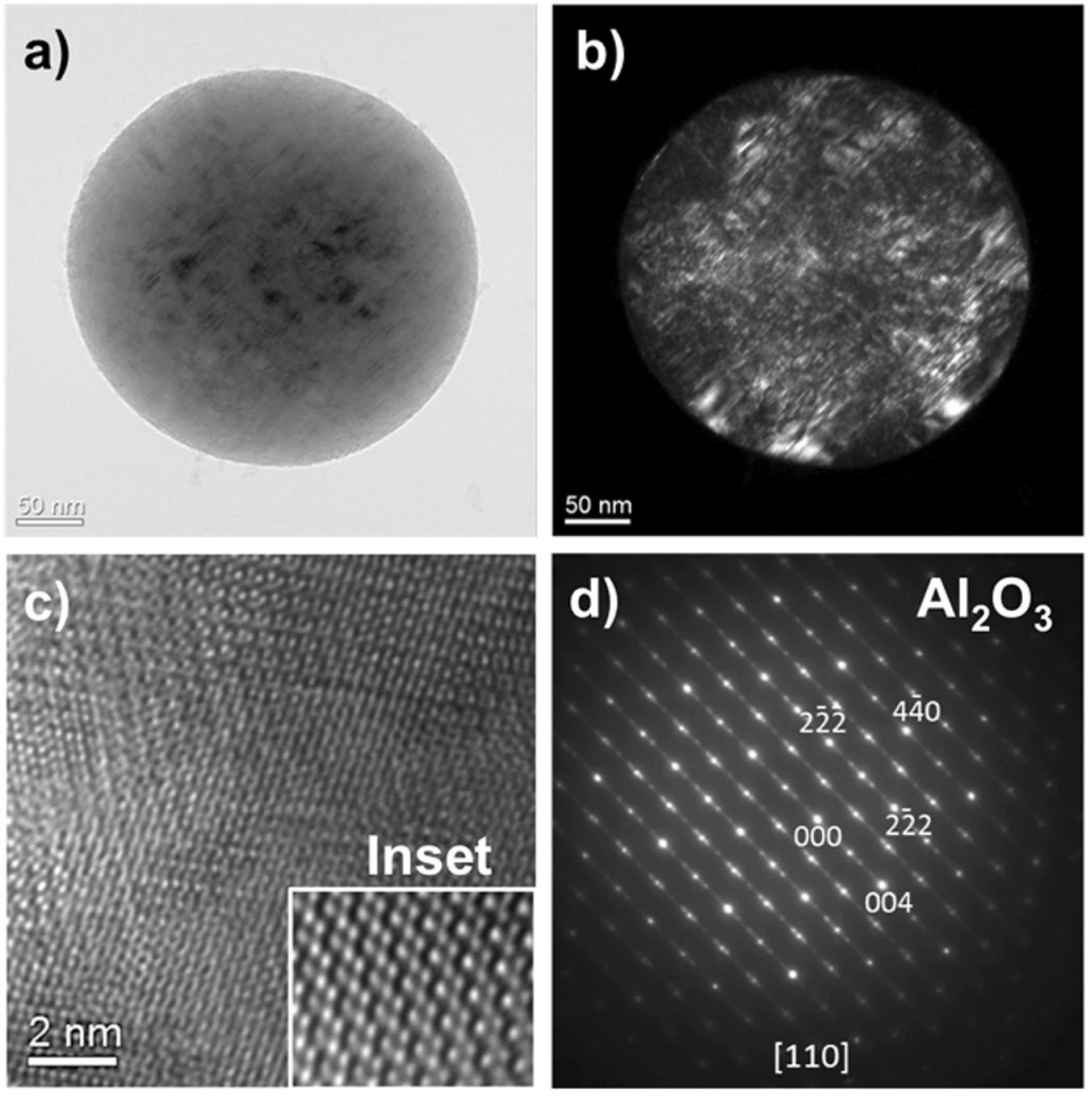
TEM images and electron diffraction pattern of spherical Al_2_O_3_ condensate collected on a carbon film: (**a**) bright-field image, (**b**) dark-field image, (**c**) high-resolution TEM image (the inset is the enlarged image), and (**d**) selected-area electron diffraction pattern (eta-Al_2_O_3_). Plane defects (e.g. micro-twins or stacking faults) can be identified in (**b**).

Bright- and dark-field TEM images of the aluminum oxide particles collected on a carbon film are shown in Fig. 8(a) and (b), respectively. The aluminum oxide particle is a ~300 nm sphere. Tiny particles (<5 nm) attached to the sphere surface are also observed. The spherical particle appears to be a single crystal based on the electron diffraction data and has a very good crystallinity. Planar crystal defects (e.g. micro-twins or stacking faults) can be identified in the sphere, as shown in the dark-field TEM image. A high resolution TEM image and a selected-area electron diffraction pattern (SADP) are shown in Fig. 8(c) and (d), respectively. The strong reflections in the SADP are indexed matching [110] zone of eta-Al_2_O_3_ crystal structure (cubic/Fd3m/a = 7.906 Å)^26^. The weak streaks and satellite reflections are attributed to the planar crystal defects that occur parallel to {100} crystal planes of the eta-Al_2_O_3_. It seems that distribution of the planar defects tends to be ordered to form a super-lattice structure. The sizes of the spherical Al_2_O_3_ particles are on the order of hundreds of nanometers, typically between 200 and 500 nm based on our TEM observations. In addition, Al_2_O_3_ spheres that are up to 2 µm in diameter have been found in SEM observations, as discussed in section 3.7.

### Uranyl Oxide Results

A large number of atomic transitions and narrow diatomic emission spectra were noted in the literature^24,27–31^ for uranium and its monoxide, resulting in a continuous spectrum that is difficult to deconvolute and infer the chemical evolution. Different experimental methodologies have been adopted in the literature to accurately distinguish the electronic transitions from each other. Use of Fabry–Perot etalon integrated with a Czerny–Turner spectrometer was shown to achieve high spectral resolutions (e.g. spectral line widths of 10 pm) to differentiate ^238^U from ^235^U and thus to determine the uranium enrichment level^32^. Another recent study^33^ used different oxygen isotopes (^16^O and ^18^O) to unambiguously identify the emission feature observed in laser ablation studies at ~593.6 nm as due to UO specifically. In the current study, we used this spectral data as a reference to compare the strong emission intensities of the uranium atom at 591.5 nm with the uranium monoxide emission centered at 593.6 nm.

**Figure 9:**
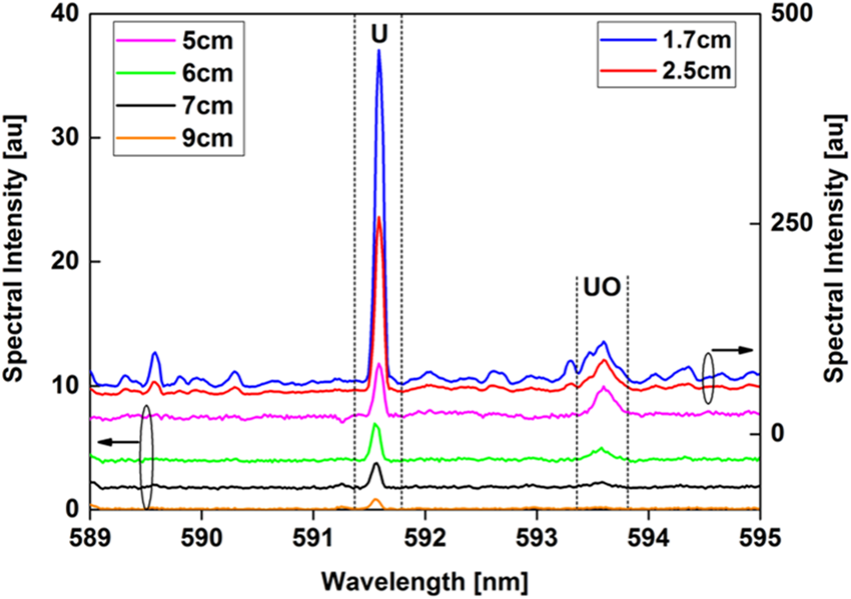
Emission spectra of atomic uranium and molecular uranium monoxide taken at different locations along the reactor. Both UO and U emission intensities decrease with distance along the reactor, but their rates of change differ. The intensities of measurements taken close to the RF coil (x: 1.7 and 2.5 cm) are plotted on the right y-axis, whereas the others are shown on the left y-axis.

**Figure 10:**
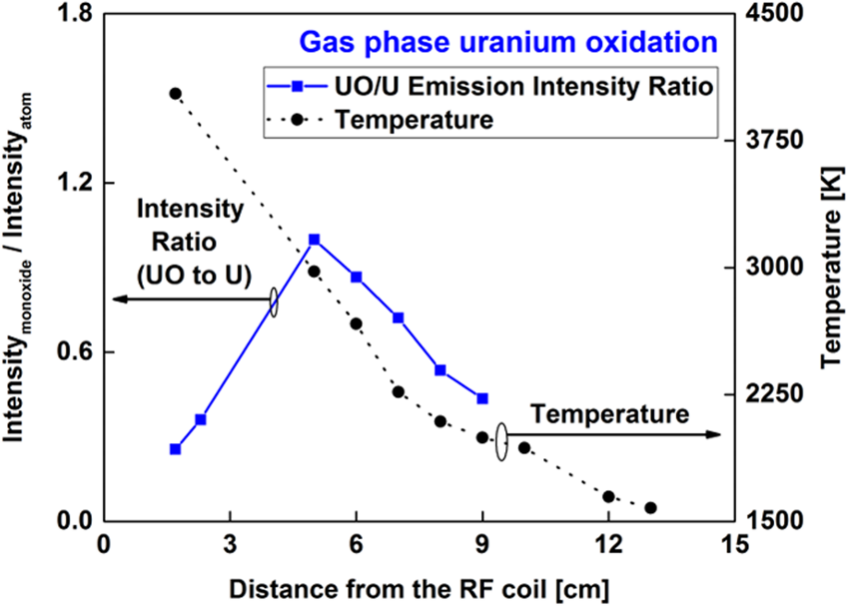
Variation of molecular-to-atomic emission intensity ratio (I_UO_/I_U_) as a function of distance along the flow reactor (left y-axis), showing the chemical evolution from U atoms to UO molecules. Temperature is shown on the right y-axis.

The emission spectra of neutral uranium atoms and uranium monoxide molecules recorded at various locations along the reactor are shown in Fig. 9. Uranium atoms have a strong emission line at 591.5 nm, whereas the UO emission is centered at 593.6 nm. The area below the UO emission spectral peak profile was integrated between 593.38 and 593.93 nm and divided by the line intensity of the U atom at 591.5 nm. The variation of molecular-to-atomic emission intensity ratio as a function of distance along the flow reactor is plotted in Fig. 10. Temperature is plotted in the same figure. The UO/U intensity ratio reaches its maximum value at 5 cm downfield from the RF coil, where the temperature was 3000 K. After that point, the relative UO/U intensity ratio decreased, presumably due to the formation of a higher uranium oxide. Gas-phase UO_2_ and UO_3_ are expected to form in an oxidizing environment at elevated temperatures^34–36^.

**Figure 11:**
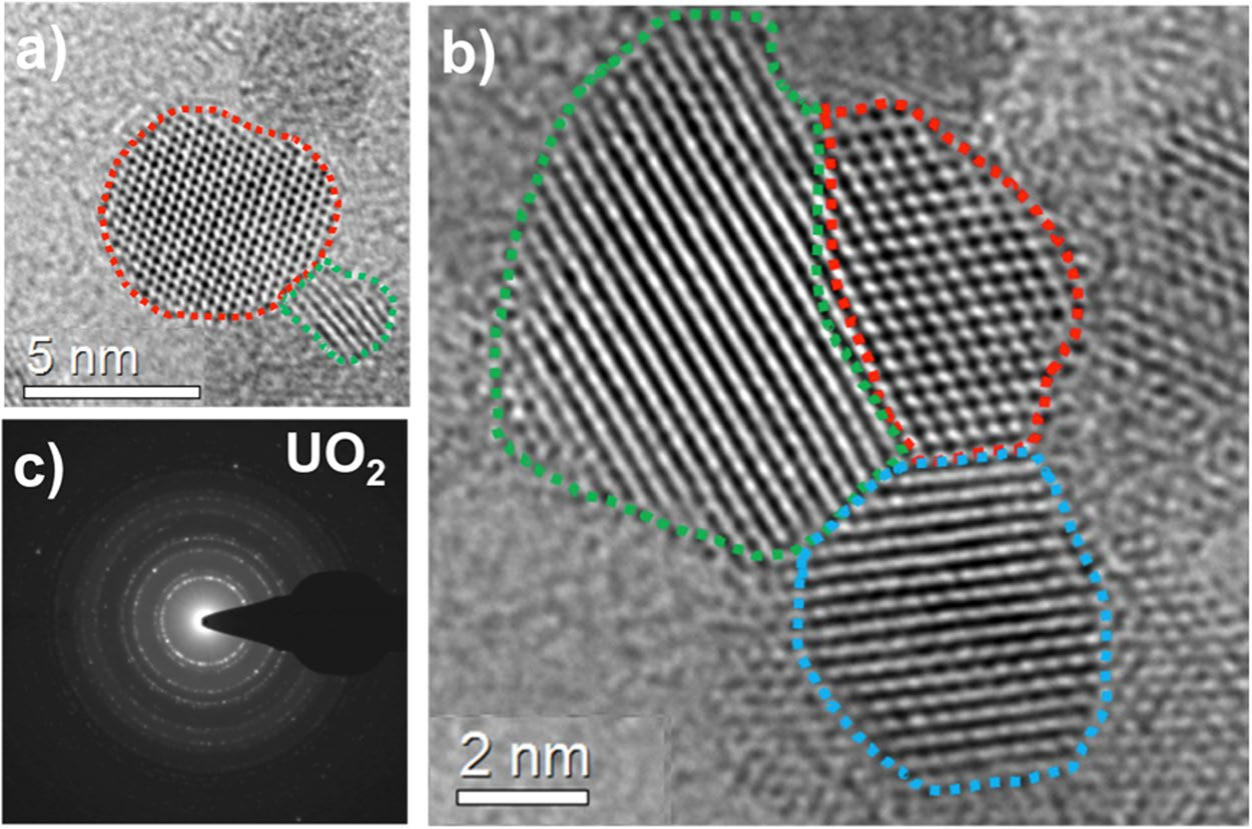
(**a**) High-resolution TEM images of UO_2_ particles collected on a carbon film. Individual UO_2_ crystals ranging in size from 2 nm to 8 nm are shown in (**a**,**b**). Dashed lines are drawn around the ordered structure of crystals to clearly identify the nano-particles. Selected-area electron diffraction pattern is given in (**c**), matching the crystal structure of fcc UO_2_.

Uranium oxide particles were collected on carbon and silicon nitride films and analyzed using transmission electron microscopy. High-resolution TEM images of the uranium oxide particles collected on a carbon film are shown in Fig. 11(a,b). The sizes of the crystalline nano-particles were between 2 and 8 nm. A corresponding selected-area electron diffraction pattern is given in Fig. 11(c). The sharp ring pattern matches the crystal structure of fcc UO_2_.

The TEM images of the uranium oxide particles collected on a silicon nitride film are given in the supplementary material (See Fig. S4). The electron diffraction pattern of the particles matches a mixture of UO_2_ and α-UO_3_ (or hexagonal U_3_O_8_). Since α-UO_3_ and hexagonal U_3_O_8_ could not be unambiguously differentiated using the diffraction pattern analysis, we also performed *in-situ* line of sight infrared absorption spectra measurements using our flow reactor and a Fourier Transform Infrared Spectrometer (FTIR)^37^. The measured infrared spectrum is given in the supplementary material (see Figs S5 and S6). Two broad absorption features were observed around 777 and 895 cm^−1^. The spectral positions and intensities of the features are similar to those reported in the literature for α-UO_3_ ^38^. Note that U_3_O_8_ has a strong absorption near 740 cm^−1^ ^38^, which was not observed in this study. Furthermore, the colors of the oxides can qualitatively help differentiate their compositions. U_3_O_8_ and UO_3_ colors are reported as dark green-black and orange-yellow, respectively^39–41^. We observed accumulation of yellow material on the walls of the quartz tube. These various lines of evidence are all consistent with the formation of α-UO_3_.

When a carbon film was used in place of the silicon nitrate, UO_3_ crystals were absent and only UO_2_ was observed. Several studies in the literature^42–44^ discussed the effect of carbon on the reduction of uranium trioxide to uranium dioxide. Poncet *et al*.^42^ studied carbon-reduction of UO_3_ particles at temperatures as high as 1073 K. They indicated that UO_3_ particles were converted to U_3_O_8_ at first, and then to UO_2_ under slow heating rates (5 K/min). Del and Wheel^44^ demonstrated the rate at which UO_3_ is reduced to UO_2_ in the presence of carbon monoxide gas is strongly dependent on temperature. The reduction rate was increased by an order of magnitude (from 100 to 10 minutes) in a platinum reactor tube when the furnace temperature was increased from 700 to 1000 K under a CO flow rate of 0.6 l/min.

In the present work, the gas temperature at the particle collection point (x = 16 cm) was around 1100 K. Although it took only 30 ms for the reactants to reach to the collection substrate (Fig. S2 in the supplementary material), the experiments were run for 180 seconds during particle collection to allow for sufficient accumulation of the oxide nanoparticles. This may have been adequate time for UO_3_ particles to undergo local reduction to UO_2_, concurrent with oxidation of the carbon substrate to form CO.

### Comparison of Results and the Effect of Oxygen Fugacity

**Figure 12:**
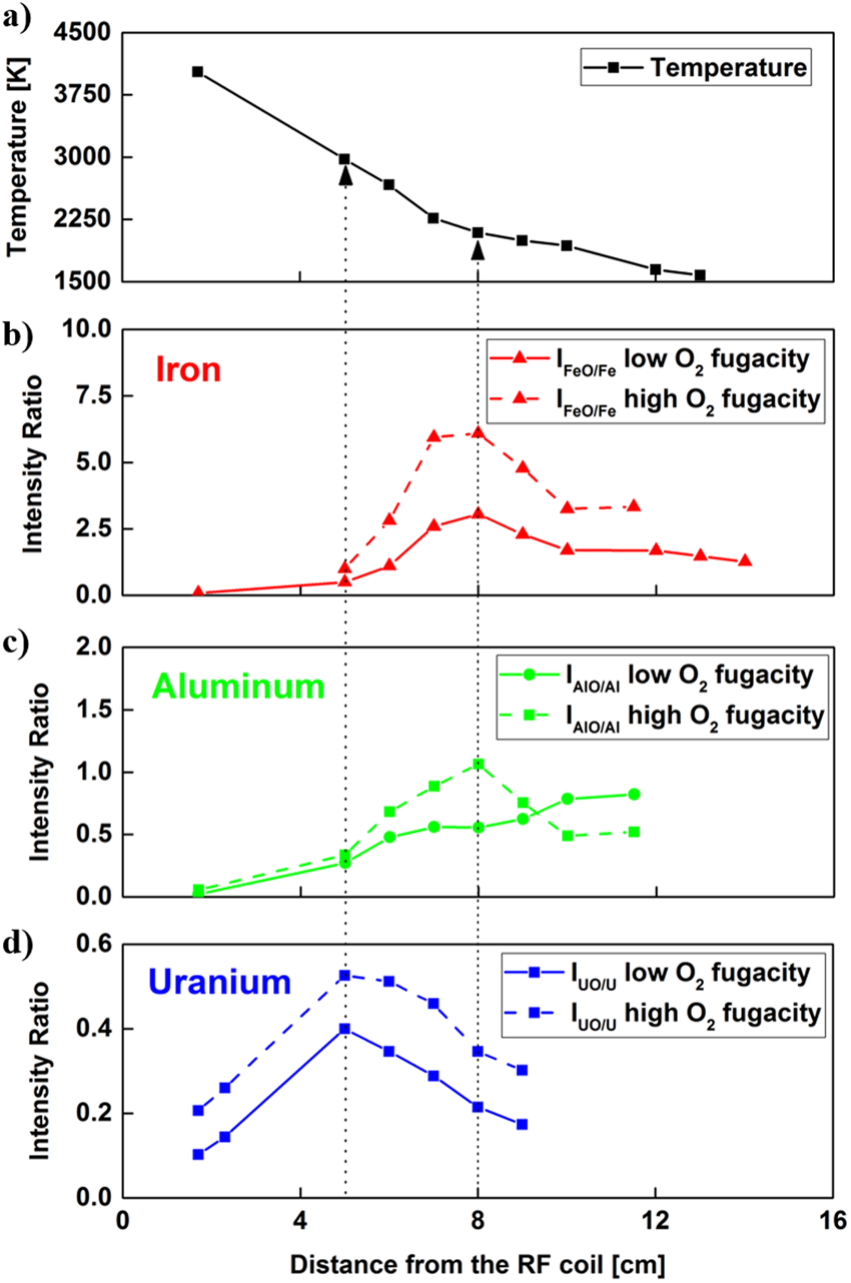
Temperature distribution along the reactor (**a**) in comparison to molecular-to-atomic emission intensity ratios for (**b**) iron, (**c**) aluminum, and (**d**) uranium. Solid and dashed lines are used to present experimental results obtained at low oxygen fugacity (experiments 1, 2, and 3 in Table 1) and high oxygen fugacity (experiments 4, 5, and 6 in Table 1), respectively.

The effect of increasing the input concentration of oxygen in the flow reactor was investigated for all three types of metal oxides. Emission spectra were taken at the same locations as those shown in Figs 4, 7 and 10 and the same method was used to obtain the emission intensity ratios as a function of distance along the reactor. Temperatures from the atomic emission of iron were also calculated based on the relative populations of line intensities and we saw that the increase of input concentration of oxygen did not change the temperatures outside the uncertainties of the measurements. The temperatures and molecular-to-atomic emission intensity ratios for three different metals are plotted together in Fig. 12. The data taken without additional oxygen are shown as solids lines (experiments 1, 2, and 3 in Table 1), whereas dashed lines represent the data taken with additional oxygen gas input (experiments 4, 5, and 6 in Table 1). The monoxide/atom intensity ratios shifted upward for all three chemical elements (Fig. 12(b–d)) due to an increase in monoxide concentrations. It appears that the location of the peak intensity ratio for iron shifted slightly upstream in the reactor, toward higher temperatures. The increase in gas phase iron monoxide molecules at higher oxygen concentrations may have caused an increase in the nucleation rate to form FeO (liquid) droplets. As a result, the plateau is reached at 7 cm rather than 8 cm. Furthermore, at high oxygen fugacity we observed that the optical emission intensities of iron atoms approached zero (SNR <5) further upstream at the 11.5 cm location. This is also due to the increased rate of formation of FeO (g) molecules at higher oxygen concentrations.

The shape of the measured intensity ratio for aluminum (Fig. 12(c)) changed substantially as the oxygen concentration was increased. The ratio of emission intensities shows a peak similar to the results of experiments obtained using iron and uranium. The oxidation of aluminum has been previously studied^45,46^ and various reactions are noted for the aluminum-oxygen system. Huang *et al*.^45^ developed a reaction mechanism that considers the formation of Al_2_O_3_(l) from AlO molecules through several steps involving other species such as Al_2_O_2_, AlO_2_ and Al_2_O. Note that our recovered particles were identified as Al_2_O_3_(s) based on the results shown in Fig. 8. Therefore, it is reasonable to expect that the rate of formation of Al_2_O_3_(l) from AlO(g) would be slower compared to the formation of FeO(l) or UO_2_(l) from their monoxides, because there are multiple reactions involved in the aluminum-oxygen system. Higher number densities of AlO molecules in the high fugacity experiment would also favor an increase in the rate of consumption of AlO to form higher aluminum oxides. This may explain the inflection in the AlO/Al intensity ratio for the high oxygen fugacity experiment.

The measured intensity ratio for uranium (Fig. 12(d)) increased by a near-constant offset between the low and high fugacity experiments up to 9 cm where the signal was no longer measurable due to the formation of higher oxides. Simulations of the kinetics of chemical reactions for uranium, aluminum, and iron are required to gain further insights into the gas phase oxidation reactions at high temperatures. In this study, a chemical kinetic model was developed to describe the oxidation of aluminum and iron metals. A chemical kinetic model for uranium oxidation was recently developed by Finko *et al*.^34^ and will be used to evaluate our experimental results for uranium in a future study.

### Chemical Kinetic Modeling

A chemical kinetic model consisting of gas-phase reaction descriptions (e.g., A + B ↔ C or A + B ↔ D + E), reaction rates, and thermodynamic properties was developed in an ANSYS CHEMKIN^47^ compatible format. Particle nucleation and growth followed the simultaneous particle and molecule modeling (SPAMM) approach developed by Pope and Howard^48^. The widely used SPAMM approach proved to be successful method for simulating multi-phase reacting systems, including modeling metal nanoparticle synthesis and in transportation related fuels to predict soot formation^49,50^.

Reaction rates for aluminum and iron submodels were taken from literature measurements and ab initio calculations when available. In the absence of known reaction rates, reversible reactions were written in their exothermic direction with a rate constant of 1.66 × 10^−10^ cm^3^/molecule/s, which is typical rate for “fast”, collision limited, exothermic bimolecular reactions in hydrocarbon systems^51^. For irreversible reactions describing the formation of particles, 30% of the theoretical bimolecular hard-sphere collision rate^45^, k_i,j_, with no additional activation energy, was assigned.

1 $${k}_{i,j}={N}_{A}{({d}_{i,j})}^{2}\sqrt{\frac{8\pi {k}_{B}T}{{\mu }_{i,j}}}$$

In Equation 1, the bimolecular collision rate between two species *i* and *j* is calculated using the Avogadro constant *N*_*A*_, the Boltzmann constant *k*_*B*_, temperature *T*, the collision diameter *d*_*i,j*_, and the reduced mass *μ*_*i,j*_. Thermodynamic properties for the metals and their oxides are from the Burcat thermochemistry database^52^, published literature^53^, or application of group additivity principles^54^. Since aqueous nitrate solutions were used to introduce aluminum and iron into the flow reactor, the hydrogen and oxygen chemical kinetics from the work of Li *et al*.^55^ were included in the present kinetic model.

**Figure 13:**
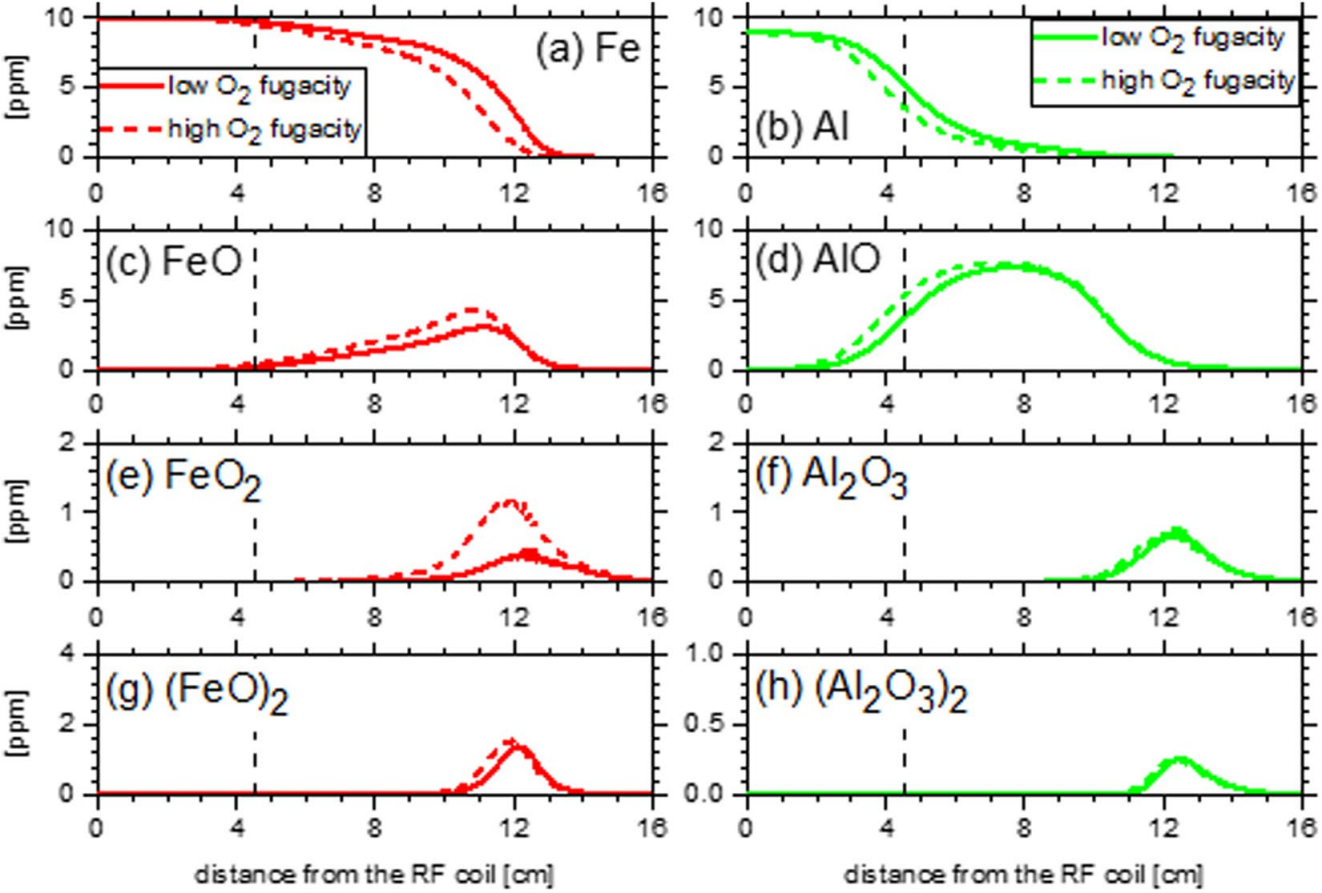
Concentrations of Fe, Al, and their predominant oxidation intermediates from coupled chemical kinetic and computational fluid dynamic simulations of experiments I, IV (left panels) and III, VI (right panels) in the plasma flow reactor. A vertical dashed line represents the location of the ring flow injector.

Computational fluid dynamic simulations employed Starccm+^56^ following the approach described in Koroglu *et al*.^13^. For this discussion, a Lagrangian approach to the chemical kinetic modeling was applied. This approach is considered adequate given the abundance of oxygen in the experiments performed and the assumption that the chemical reaction time scales are sufficiently faster than the transport phenomena. The 0-D homogeneous closed reactor module of CHEMKIN with constrained pressure and temperature was used to model experiments I, III, IV, and VI of Table 1. Temperature as a function of residence time (Fig. S2) was specified using the history of a representative parcel of gas from the computational fluid dynamic simulation flow field. Compositions from experiments I, III, IV, and VI in Table 1 were used for initial conditions of the kinetic modeling. Predicted concentrations of species as a function of time from the CHEMKIN simulation were transformed back to an axial distance via a coordinate transformation informed by the CFD simulation. Figure 13 presents modeling results based on this approach.

Concentrations of iron (Fig. 13a) and aluminum (Fig. 13b), as well as their oxides (Fig. 13c–h) from the coupled kinetic and CFD simulations show a sensitivity to the kinetic pathways which lead to stable oxides and the initial oxygen fugacity of the experiments. Iron consumption and iron oxide production are unperturbed by the oxygen fugacity close to the RF coil, <4 cm, where the temperature exceeds 3000 K. However, once the flow has progressed to and beyond the location of the ring flow injector (4.5 cm) significant changes in Fe (Fig. 13a) and FeO (Fig. 13c) are observed in agreement with experimental observations. FeO is the first iron oxide formed via the termination reaction Fe + O ↔ FeO (ΔH = −414 kJ mo^−1^), and the first stable iron intermediate which contributes to its longevity in the flow reactor. Concentrations of FeO remain higher than 1 ppm for up to 12.5 cm past the RF coil. Experimental results in Fig. 12b show that FeO emission is last measured in the 12–14 cm region, indicating that the current kinetic model reasonably captures the long lifetime of FeO in the flow reactor. In both the model and experiments, the first aluminum oxide (the radical AlO, Fig. 13d), is formed and consumed over a broad length of the flow reactor. Production of AlO is from the initial abundance of Al and O atoms via the reaction Al + O ↔ AlO (ΔH = −511 kJ mol^−1^). The model agrees reasonably with the experimental results presented in Fig. 12c that indicate AlO has likely been effectively consumed by 12 cm. For both the iron and aluminum experiments, the model captures the effect of the higher initial atomic oxygen fugacity which promotes faster formation of the initial oxides.

Differences in the location of the first stable iron oxide (Fig. 13c) and aluminum oxide (Al_2_O_3_, Fig. 13f) from the model highlight the considerable influence kinetics may exert prior to aggregation and agglomeration processes. Formation of Al_2_O_3_ proceeds through a multi-step reaction process that includes the species Al, AlO, AlO_2_, Al_2_O, and Al_2_O_2_, which act as barriers slowing the formation of a stable aluminum oxide. Formation of AlO_2_ from AlO and atomic oxygen is the preferred oxidation step of AlO. However, AlO_2_ functions as an intermediate reservoir, as AlO_2_ quickly dissociates to Al atoms and molecular oxygen at the elevated temperatures of the flow reactor. As a result, AlO persists until the formation of Al_2_O, which can then undergo further reaction with the available atomic oxygen. Current kinetic modeling efforts indicate that the formation of stable Al_2_O_3_ is dominated by the radical termination of Al_2_O_2_ + O ↔ Al_2_O_3_, ΔH = −393 kJ mol^−1^. For the experiments considered (Table 1), atomic oxygen is abundant and the concentration of Al_2_O_2_ radicals is therefore the rate limiting factor.

After the stable aluminum oxide is formed, the clustering and formation of larger aluminum oxide particles such as (Al_2_O_3_)_2_ (Fig. 13h) proceeds quickly. This rapid clustering of Al_2_O_3_ is enabled by the relatively high stability of the larger particles, where the enthalpy of formation of Al_2_O_3_ is −547 kJ mol^−1^, −1929 kJ mol^−1^ for (Al_2_O_3_)_2_, −3314 kJ mol^−1^ for (Al_2_O_3_)_3_, and −4660 kJ mol^−1^ for (Al_2_O_3_)_4_ ^53^. While the molar concentrations of (Al_2_O_3_)_n,_ where n ≥ 2, particles are typically low (<1ppm) in the model, these particles contain significant fractions of the initial aluminum introduced to the flow reactor. Unlike larger aluminum oxides, the initial formation of larger iron oxides is predicted to be slower in the current kinetic model. The difference between the peak locations of (FeO)_2_ in Fig. 13g and FeO in Fig. 13c is bigger than the one between Al_2_O_3_ in Fig. 13h and (Al_2_O_3_)_2_ in Fig. 13f. The current kinetic model is built assuming that the larger (n ≥ 2) iron oxides possess relatively higher enthalpies of formation, and are therefore less stable, than larger aluminum oxides based on available binding energies for both metal oxides^53,57^, which leads to the slower initial growth of large iron oxides.

### Morphology and Size of Particles

**Figure 14:**
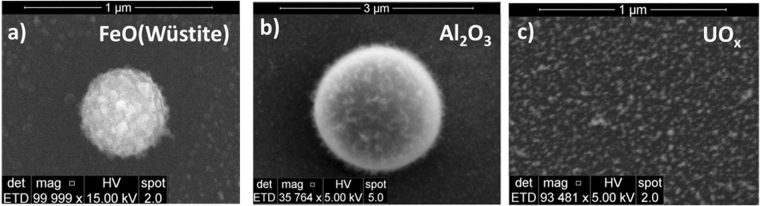
Comparison of secondary electron SEM images for (**a**) iron, (**b**) aluminum, and (**c**) uranium oxides. Particles were collected on silicon wafers.

The high-resolution TEM images shown in Figs 5 and 11 enabled us to identify the individual crystals of metal oxides, which ranged from 2 to 8 nm for uranium oxides and 5 to 30 nm for iron oxides. The single crystals of spherical aluminum oxide particles were much bigger (200 to 500 nm). In this study, particles were also collected on bare silicon wafers located 16 cm from the RF coil to observe the morphology and aggregate sizes of different metal oxides. SEM images of uranium, aluminum, and iron oxide agglomerates are shown in Fig. 14. Al_2_O_3_ particles are up to a couple of microns in size, whereas iron oxides reach agglomerate sizes up to several hundred nm. Uranium oxide particles have a maximum agglomerate size under 100 nm. In addition, aluminum and iron oxide agglomerates tend to be spherical in form, whereas uranium oxides form irregular aggregates. The x-ray energy dispersive spectra of the samples indicated that the silicon substrates were entirely covered with metal oxides. We also observed a particle size distribution on each substrate. For example, spherical droplets of Al_2_O_3_ varying in sizes from a couple of hundred nanometers to microns were seen. However, such large particulates were never observed for uranium oxides.

The physical mechanisms of condensation during fallout formation is dependent on whether there exists a carrier material on which radionuclides such as actinides, fission and activation products (e.g. iron) can condense. Such particulates can serve as nucleation sites (heterogeneous nucleation), in which case small saturation levels would be enough for vapor molecules to condense. However, if there is no such particle readily available around vapor molecules, which is the situation in our current experiments, then an energy barrier must be exceeded to form stable embryos in the liquid phase (homogeneous nucleation). High degrees of supersaturation are required to create nuclei in the absence of an existing condensation surface, because the droplet size must be large enough to overcome the surface tension forces that could lead to break-up and evaporation. In other words, a critical particle size (or critical number of molecules in a cluster) must exist to enable the genesis of a stable embryo required for further condensation to occur.

The super-saturation level, which is the driving force for nucleation, can be defined as the ratio of the vapor pressure of the oxide, *p*(*T*) to its equilibrium vapor pressure *p*_*e*_(*T*)

2 $$S=\frac{p(T)}{{p}_{e}(T)}$$

Equilibrium saturation pressures, *p*_*e*_(*T*), as a function of temperature can be derived using the Clasius-Clapeyron equation

3 $$\mathrm{ln}\,\frac{{p}_{e}(T)}{{p}_{e}({T}_{0})}=\frac{{\rm{\Delta }}{h}_{fg}}{{R}_{u}}(\frac{1}{{T}_{0}}-\frac{1}{T})$$

where *p*_*e*_*(T*_0_) is the equilibrium saturation pressure at some reference temperature *T*_0_, which can be taken as the boiling point to calculate the variation of saturation pressure, p_e_, as a function of temperature, *T*, using the latent heat of vaporization Δ*h*_*fg*_ and universal gas constant *R*_*u*_. The values of boiling (and melting) temperatures and latent heat of vaporization (and fusion) are summarized in Table 2.

**Table 2:** The latent heat of vaporization and fusion and melting and boiling temperatures of FeO, Al_2_O_3_, and UO_2_^14^.

	${\boldsymbol{\Delta }}{{\boldsymbol{h}}}_{{\boldsymbol{fusion}}}$ [kJ/mole]	${{\boldsymbol{T}}}_{{\boldsymbol{melting}}}$ [K]	${\boldsymbol{\Delta }}{{\boldsymbol{h}}}_{{\boldsymbol{vaporization}}}$ [kJ/mole]	${{\boldsymbol{T}}}_{{\boldsymbol{boiling}}}$ [K]
FeO	31	1647	230	2785
Al_2_O_3_	113	2319	486	3253
UO_2_	136	3113	624	4373

**Figure 15:**
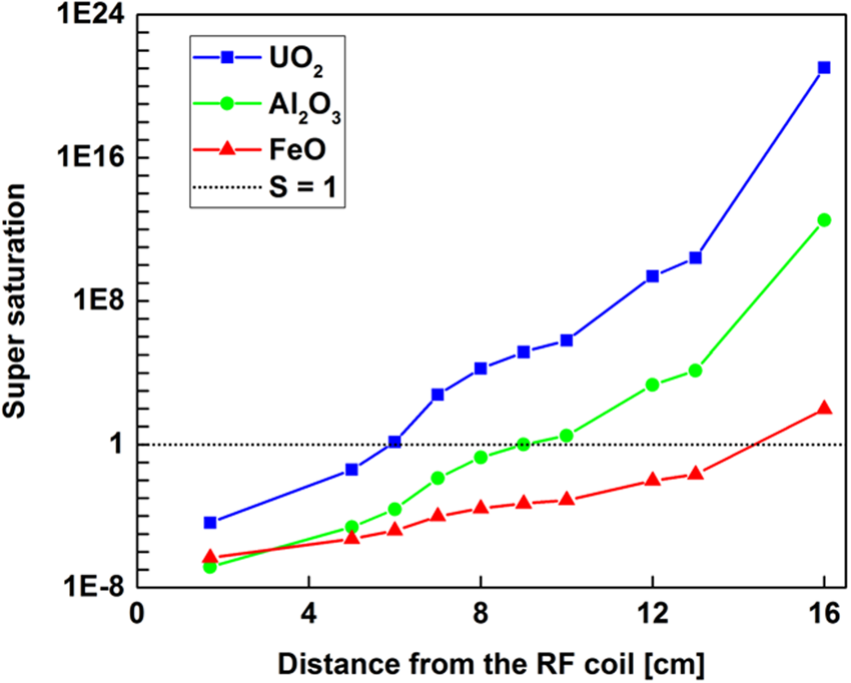
Variation of calculated super-saturation levels for three metal oxides as a function of distance along the reactor.

Temperature distribution and concentrations of metal oxides in the gas phase are required for calculating the supersaturation values as a function of position along the reactor. Using the temperature data obtained from experimental measurements and assuming the input metal concentrations given in Table 1 for iron, aluminum, and uranium are stoichiometrically converted to their oxides starting from x = 0, we plotted the super saturation values for FeO, Al_2_O_3_ and UO_2_ as displayed in Fig. 15. Supersaturation rates increase after x = 4.5 cm due to the rapid cooling of gas, coincident with volume expansion in the flow reactor due to the ring flow injector. The supersaturation (S > 1) for the most refractory oxide (i.e. UO_2_) occurs first with a steep rise, followed by the less refractory aluminum and iron oxides, in accordance with the oxide boiling temperatures (Table 2).

When the supersaturation increases, the nucleation rate increases as well, leading to an increased number of small particles. Therefore, small particles are expected to form for a refractory oxide such as UO_2_, where the degree of supersaturation increases rapidly over a short distance along the flow reactor axis. In comparison, the slower rate of nucleation and growth via condensation for FeO tends to favor the formation of fewer numbers of larger particles. Our experimental observations (TEM and SEM images) for the particle sizes of uranium and iron oxides are consistent with this logic. However, aluminum oxide exhibits a different phenomenon: single crystals are formed that are significantly larger than those observed for either uranium or iron. This could be due to the kinetically limited processes as discussed in section 3.6 for aluminum oxidation. In other words, several reaction steps involved in the complete oxidation of aluminum results in the formation of Al_2_O_3_(g) downstream of the reactor thus slowing down the nucleation and growth of the particles and resulting in small number of large single crystals. Similar sizes of alumina particles were reported by Whyman^58^ in his study on condensation of refractory oxides condensed from thermal plasma vapors. The growth of alumina crystals was attributed to the reactions of aluminum and oxygen atoms together with other sub-oxide molecules (e.g. AlO, Al_2_O_2_, etc.) on the crystal face. Electrostatic effects are also noted in the literature for the formation of large aggregates of spherical alumina particles^59^. However, we have not observed aggregates of alumina nanoparticles for our experimental conditions.

In the present study, two types of nucleation processes could precede condensation: (1) homogeneous nucleation of new embryos and (2) heterogeneous nucleation on preexisting particles such as ionized species in the plasma. Ion-induced heterogenous nucleation can increase the effective supersaturation level, leading to an energetically favored nucleation path^60^. However, the volume averaged number density of the ions account for only 0.02% of the total number of atoms in thermal plasmas including ICPs^58,61,62^. This value would be even smaller at atmospheric pressures (due to rapid thermalization by increased number of collisions)^63,64^ and downstream of the ICP where FeO and Al_2_O_3_ nucleation occurs (T ~ 2500 K). Since nucleation rate is directly proportional to number densities^65^, improvements in the nucleation rate due to the presence of ions will be orders of magnitude smaller than that of the homogenous nucleation. For the same reason, the ionized species are not included in our chemical kinetics model, because their reaction rates are much lower than those of the neutral ones. However, ionized species may play an important role in the oxidation and nucleation of uranium oxides, since uranium oxidation and nucleation occurs at higher temperatures (T > 4000 K)^34^ in closer proximity to the argon plasma source. The modeling of those processes will be the scope of a future study.

## Conclusions

In this study we obtained experimental data to advance our fundamental understanding of gas phase chemical reactions of three different metal oxides and of the resulting chemical and physical properties of condensed and solidified particulates. Plasma flow reactor experiments were conducted at temperatures between 5000 K and 1000 K, atmospheric pressures, and relatively fast cooling rates (∆t < 30 ms). Analytes were input to the plasma as aqueous solutions of iron, aluminum, and uranyl nitrates using a nebulizer with argon carrier gas. The evolution of chemistry from atomic species (e.g. U, Al, Fe) to molecules (e.g. UO, AlO, FeO) was investigated as a function of temperature and oxygen fugacity using optical emission spectroscopy. The recovered oxide particles were analyzed *ex situ* using scanning and transmission electron microscopy.

Electron diffraction patterns of the recovered particles enabled us to show that gas phase iron monoxide condensed and solidified into FeO (wustite), whereas aluminum monoxide molecules formed eta-Al_2_O_3_ crystals. UO_2_ and/or UO_3_ crystals were formed depending on the type of substrate used to recover the particles, highlighting the importance of local redox conditions on the resulting form of the uranium oxides. Furthermore, a substantial difference in the particle sizes among different metal oxides was observed, even though the experimental conditions were held constant (i.e. input number densities of metals and temperature distribution along the reactor). Rapid cooling at high-temperatures resulted in the formation of small and irregular refractory UO_2_ crystals (2–10 nm), whereas the most volatile metal oxide investigated in this work, iron oxide, formed spherical aggregates (up to 500 nm) comprised of small FeO (wustite) crystals (5–30 nm). We observed spherical single crystals of eta-Al_2_O_3_ particles as large as 500 nm, consistent with a slow rate of nucleation leading to few numbers of large particles. A chemical kinetic model was developed to assist our interpretation of the experimental data. The model predicts that the nucleation of alumina will be slow due to the formation of Al_2_O_3_(g) through a multi-step reaction process. This suggests Al_2_O_3_ particles form larger single crystals because it is energetically more favorable to add material to an existing crystal compared to nucleating and growing new crystals under these experimental conditions.

This work offers insights into fallout formation mechanisms by investigating the roles of (1) gas phase reaction kinetics, (2) physical mechanisms of particle formation through nucleation and condensation processes, and (3) local redox conditions, on the resulting sizes and phases of the nanoparticles. As a future work, we will investigate the effects of varying cooling time scales and input number densities of metals.

### Data availability

The datasets generated during and/or analysed during the current study are available from the corresponding author on reasonable request.

## Electronic supplementary material

Supplementary Material
