# Supplementary material for: Gas Phase Chemical Evolution of Uranium, Aluminum, and Iron Oxides
Source: Sci Rep. 2018 Jul 11;8:10451. doi: 10.1038/s41598-018-28674-6 (PMC6041320; doi:10.1038/s41598-018-28674-6)
Supplement: Supplementary file 1 — Supplementary Material [file 41598_2018_28674_MOESM1_ESM.docx]

SUPPLEMENTARY MATERIAL

**Gas Phase Chemical Evolution of Uranium, Aluminum, and Iron Oxides**

Batikan Koroglu *, Scott Wagnon, Zurong Dai, Jonathan Crowhurst, Michael R. Armstrong, David Weisz, Marco Mehl, Joseph M. Zaug, Harry Radousky, Timothy Rose

*Physical and Life Sciences Directorate, Lawrence Livermore National Laboratory, Livermore, California, 94550, USA*

****Corresponding Author: koroglu1@llnl.gov***

1. **Comparison of Temperature determined from emission of Fe atoms and AlO molecules**

We performed temperature measurements using Fe and AlO emission. The results are shown in Figure 1S. The two sets of measurements were consistent within the measurement uncertainties (+/- 10%).





Figure 1S. Temperature determined from the atomic iron and molecular aluminum monoxide emission intensities are compared. Temperatures agreed within the uncertainty of the measurements.

1. **Modeled temperature distributions plotted as a function of location and residence time**

The modeled temperature is plotted as a function of distance (bottom x-axis) and residence time (top x-axis) in Figures 2S, which is obtained from the computational fluid dynamics (CFD) model.





Figure 2S. Variation of temperature along the reactor as a function of location (bottom x-axis) and residence time (top x-axis) obtained from the computational fluid dynamics simulation.

1. **TEM images of iron oxide particles collected on a silicon nitride film**

The TEM images of iron oxide particles collected on a silicon nitride film are displayed in Figure 2S. Crystalline FeO (wüstite) particles were observed.


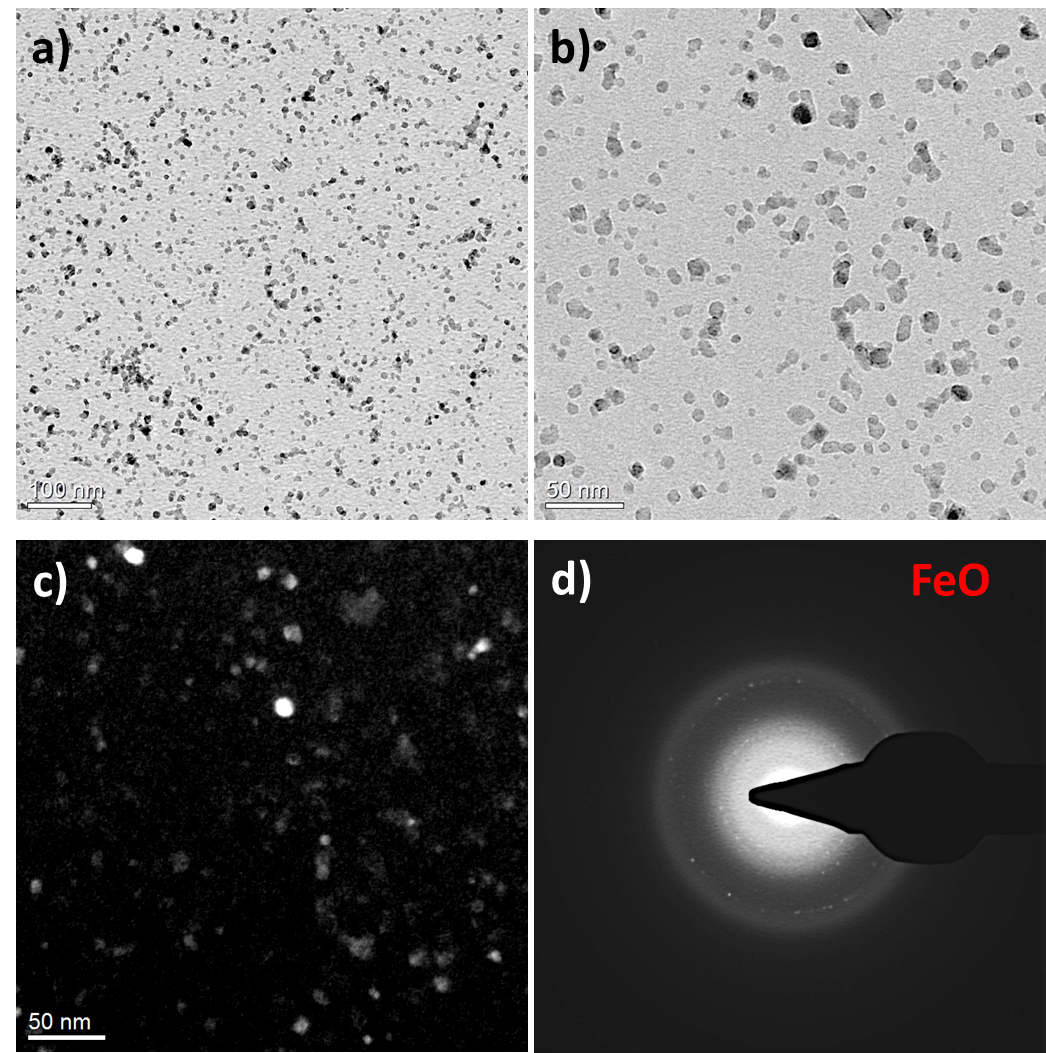


Figure 3S. TEM images of FeO particles: (a) and (b) Bright-field TEM images, (c) Dark-field TEM image, (d) selected-area electron diffraction pattern. FeO (wüstite) was the phase of the oxide formed.

1. **TEM images of uranium oxide particles collected on a silicon nitride film**

The TEM images of the uranium oxide particles collected on a silicon nitride film are shown in Figure 3S. Both UO_2_ and UO_3_ were observed.


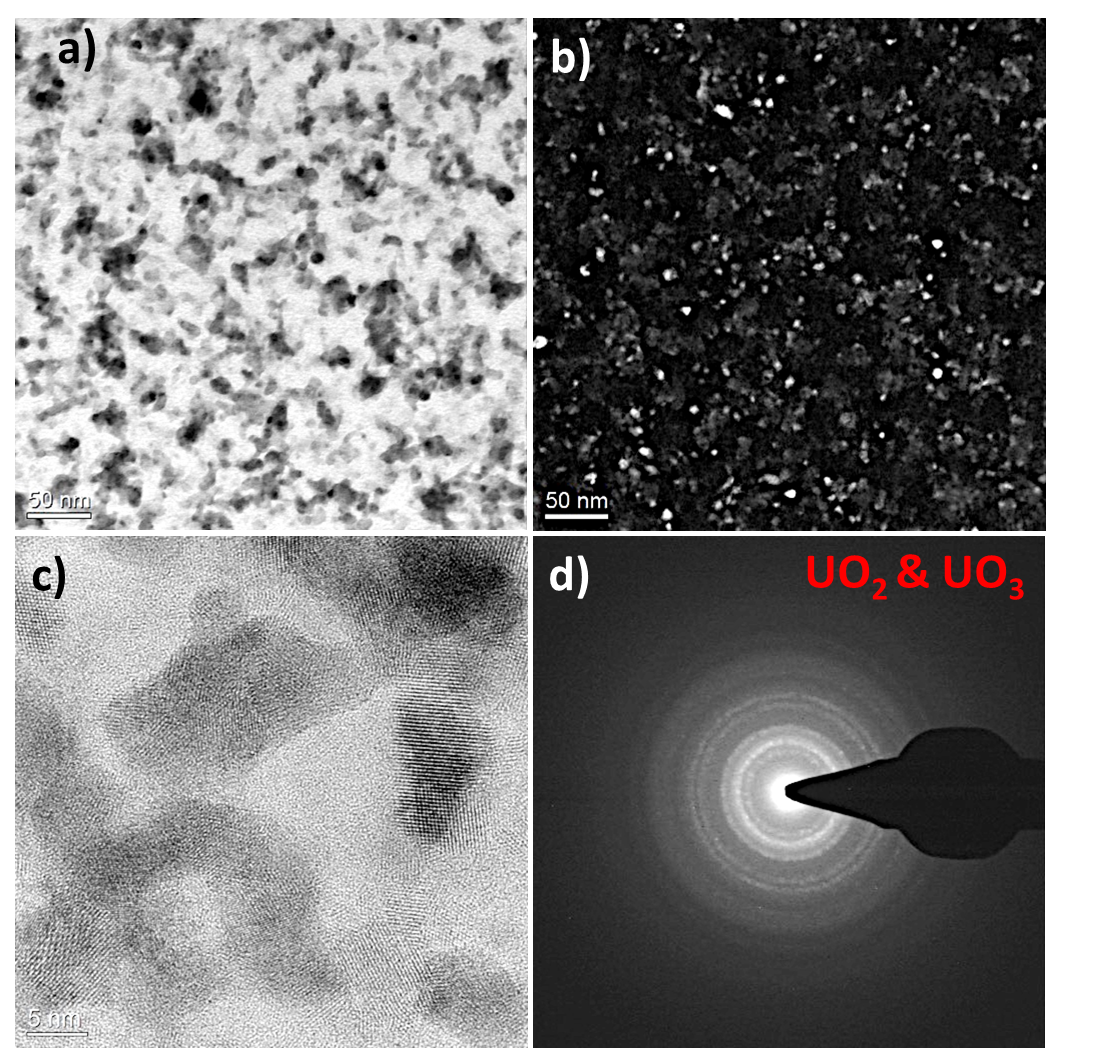


Figure 4S. TEM images of UO_2_ and UO_3_ particles observed on a silicon nitride film: (a) and (b) bright and dark-field TEM images, (c) selected-area high resolution TEM image, and (d) selected-area electron diffraction pattern

1. **Infrared Absorption Spectra of solid UO3**

Absorption spectrum of uranium oxide was measured using the globar light source from a Bruker FTIR (Vector-33) and our flow reactor setup. Since our flow reactor is made up of quartz which is not transmissive at low wavenumbers, we have designed and built another similar flow reactor with ZnSe windows transmissive up to 16 µm. The details of this flow reactor design will be discussed in a future publication. A simple schematic of the line-of-sight measurements is given in Figure 4S. The ZnSe windows are attached to a quartz tube by means of Swagelok ultra-torr fittings.


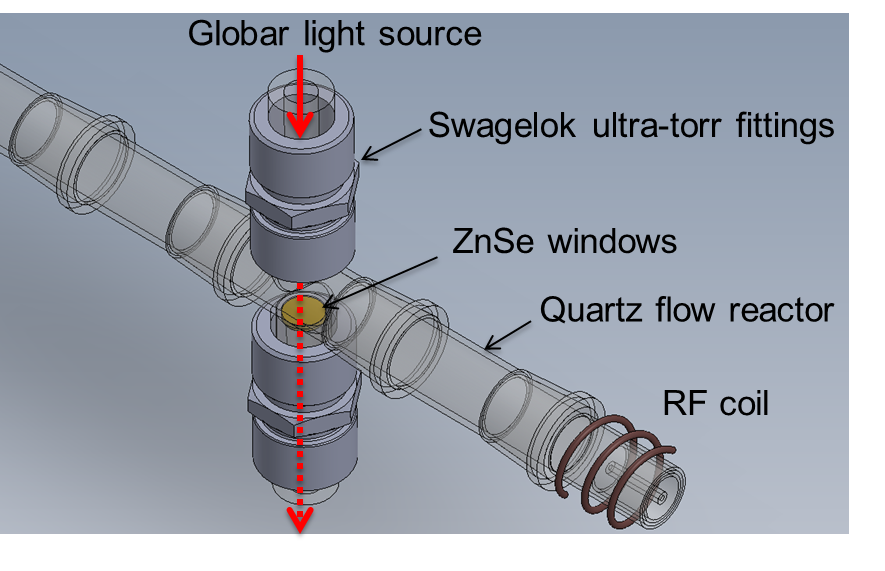


Figure 5S. Flow reactor with ZnSe windows attached by means of Swagelok fittings

The spectra were recorded using a liquid nitrogen cooled MCT detector over the wavenumber range between 650 and 1200 cm^-1^. The configuration of the FTIR is given in Table 1S. Uranium oxide spectrum is shown in Figure 5S, which was calculated by Fourier transform of 200 co-added interferograms.

**Table 1S** FTIR Spectrometer Configuration

| Light source | Globar |
| --- | --- |
| Beamsplitter | Potassium bromide (KBr) |
| Detector | MCT HgCdTe |
| FTIR input aperture | 3 mm |
| Resolution | 1 cm^-1^ |
| Phase correction, Zero-filling | Mertz, 2× zero-filling |
| Apodization function | Boxcar |





Figure 6S. UO_3_ absorption spectra measured using an FTIR and our flow reactor setup
